# SARS-CoV-2-specific immunity after XBB.1.5 vaccination is not significantly altered by subsequent influenza vaccination in dialysis patients

**DOI:** 10.1038/s41598-026-60726-0

**Published:** 2026-07-06

**Authors:** Saskia Bronder, Rebecca Urschel, Felix Reinhardt, Janine Mihm, Élora Schlienger, Tina Schmidt, Susanne Brückner, Urban Sester, Martina Sester

**Affiliations:** 1 Department of Transplant and Infection Immunology, Saarland University, 66421, building 77, Kirrberger Straße, PharmaScienceHub, Homburg, Germany; 2 Dialysezentrum Saarlouis, Saarlouis, Germany; 3 Westpfalzklinikum Kaiserslautern, Kaiserslautern, Germany; 4 Arbeitsgemeinschaft Heimdialyse Saar e.V, Völklingen, Germany; 5 Center for Gender-specific Biology and Medicine (CGBM), Saarland University, Homburg, Germany

**Keywords:** Sequential vaccination, monovalent XBB.1.5 vaccine, quadrivalent influenza vaccine, dialysis, antibodies, T cells, Diseases, Immunology, Medical research, Nephrology

## Abstract

Annual immunisation against COVID-19 and seasonal influenza before the winter waves is increasingly recommended in routine practice. These vaccines may be administered on the same day or sequentially, yet data on the immunogenicity of consecutive vaccinations in patients on dialysis remain limited. In this real-world observational study, we assessed SARS-CoV-2-specific immune responses in dialysis patients receiving the monovalent XBB.1.5-vaccine followed by a quadrivalent influenza vaccine 14 days later, or either vaccine alone. Antigen-specific antibodies and T cells were quantified using enzyme-linked immunosorbent assays and flow cytometry. Baseline analyses showed that most patients had detectable SARS-CoV-2- and influenza-specific immunity prior to the vaccination season. Both vaccines substantially boosted pre-existing humoral and cellular responses. Among XBB.1.5-vaccinated patients, subsequent influenza vaccination did not alter the magnitude of spike-specific antibody or T-cell responses. Likewise, influenza vaccination had no non-specific effect on SARS-CoV-2-specific immunity. Spike-specific responses remained stable for six months and persisted at levels exceeding those of unvaccinated patients assessed during the same period. Sequential administration of COVID-19 and influenza vaccines in patients on dialysis is feasible and was not associated with compromised immunogenicity of either vaccine. These findings support the use of booster vaccination in these patients and inform future deployment of additional mRNA-based vaccines.

**Supplementary Information:**

The online version contains supplementary material available at 10.1038/s41598-026-60726-0.

## Introduction

Infectious diseases of the lower and upper respiratory tract can be caused by rhino-, corona-, (para)influenza and respiratory syncytial viruses^1,2^. In the northern hemisphere, respiratory infectious diseases occur seasonally in the fall and winter, and slowly decline after spring^3^. Patients with end-stage chronic kidney disease or dialysis treatment are at increased risk of respiratory viral infections with severe disease and serious outcome. To protect this vulnerable group from severe disease and hospitalization, the German standing committee for vaccine recommendations (STIKO) recommends annual influenza and COVID-19 booster vaccinations with vaccines adapted to the predominant variants against influenza and SARS-CoV-2^4^. Due to uremic immunodeficiency and multiple comorbidities^4^, patients on dialysis are known to show variably impaired immune responses against a variety of vaccinations such as herpes zoster^5^, influenza^6^ or COVID-19^8–10^. Nevertheless, we have recently shown that the bivalent BA.4/5 mRNA vaccine administered in the winter season 2022 induced IgG, neutralizing titers, and specific CD4 and CD8 T-cell levels in dialysis patients to a comparable extent as in immunocompetent individuals with even higher levels of CD4 T cells in patients with a history of SARS-CoV-2 infection^7^. In subsequent seasons, the Omicron subvariant XBB.1.5 and JN.1 dominated and led to an increased incidence of breakthrough infections, due to a strongly reduced neutralizing antibody activity toward these variants in both dialysis patients^8^ and healthy individuals^9,10^. Starting with the XBB.1.5 vaccine season, the STIKO recommended simultaneous administration with the quadrivalent influenza vaccine^11^, despite the fact that studies on co-administration of the novel mRNA vaccines with other standard protein-based vaccines were rare or yielded conflicting results. Some studies in health care workers and immunocompetent adults > 60 years of age showed lower antibody levels and a lower quantitative and functional antibody response after co-administration compared to a COVID-19 booster alone^12,13^, while others found that co-administration had no significant effect on COVID-19-vaccine induced immunity^14,15^. The Commission on Hygiene and Infection Prevention of the German Society of Nephrology (DGfN) recommended sequential vaccinations for patients on dialysis (distance ≥ 14 days)^16^, although it was unknown, whether a COVID-19-vaccine induced immune response was influenced by a subsequent influenza vaccination. We therefore used a real-world setting and characterized baseline immunity towards SARS-CoV-2 and influenza in patients on dialysis prior to vaccination. Moreover, we performed a detailed assessment of SARS-CoV-2- and influenza-specific immune responses in dialysis patients receiving the monovalent XBB.1.5-vaccine followed by quadrivalent influenza vaccination. Moreover, vaccine-induced SARS-CoV-2-specific immunogenicity in patients after sequential vaccination was compared with patients who only had received monovalent XBB.1.5 or quadrivalent influenza vaccination, respectively. Finally, the stability of XBB.1.5-vaccine-induced immunity was analysed over a period of 6 months.

## Results

### Study population

Fifty-six patients on dialysis were vaccinated and participated in the study (55 hemodialysis, 1 continuous ambulatory peritoneal dialysis). Information on patient characteristics including primary disease that led to renal failure resulting in dialysis treatment, time on dialysis, previous kidney transplantation, immunosuppressive therapy, SARS-CoV-2-related history and differential blood counts are shown in Table 1. The mean age of the patients was 69.1 ± 13.8 years (73.2% male, 26.8% female). Most patients had a history of homologous mRNA vaccination, and between three and up to seven prior immunisation events (including vaccinations and infections). A total of 43 patients (76.8%) had at least one immunisation event attributable to a previous SARS-CoV-2-infection.

**Table 1:** Demographic and clinical characteristics of the study population.

Characteristics	Dialysis patients
	(*n* = 56)
Years of age, mean (SD)	69.1 (13.8)
Sex, n (%)^a^	
Female	15 (26.8)
Male	41 (73.2)
Type of dialysis, n (%)	
Hemodialysis	55 (98.2)
Peritoneal dialysis	1 (1.8)
Time on dialysis (years), mean (SD)	3.9 (4.6)
Cause of kidney failure, n (%)	
Autoimmune-mediated nephropathy	5 (8.9)
Chronic glomerulonephritis	4 (7.1)
Secondary chronic renal disease	28 (50.0)
Innate	10 (17.9)
Other	6 (10.7)
Unknown	3 (5.4)
Previous kidney transplantation, n (%)	4 (7.1)
Immunosuppressive therapy^b^, n (%)	6 (10.7)
Glucocorticoids, n (%)	4 (7.1)
MMF, n (%)	1 (1.8)
Prednisolone/MMF, n (%)	1 (1.8)
Differential blood counts median (IQR) cells/µl	
Leukocytes, *n* = 41	6200 (2450)
Granulocytes, *n* = 32	4465 (2641)
Monocytes, *n* = 32	591 (318)
Lymphocytes, *n* = 32	1088 (747)
Percentage of T cells median (IQR) % among lymphocytes	*n* = 56
CD4 T cells	36.9 (14.0)
CD8 T cells	18.6 (9.5)
SARS-CoV-2-related characteristics before XBB.1.5-vaccination	
History of COVID-19 vaccination regimen, n (%)	
mRNA only	42 (75.0)
Vector/mRNA combination	3 (5.4)
Unknown	11 (19.6)
Prior SARS-CoV-2-infection^c^, n (%)	43 (76.8)
Total number of prior immunisation events^c^, n (%)	
3	4 (7.1)
4	14 (25.0)
5	17 (30.4)
6	9 (16.1)
7	3 (5.4)
Unknown	9 (16.1)
Type of previous immunisation, n (%)	
Vaccination	24 (42.9)
Infection	13 (23.2)
Unknown	19 (33.9)
Days since last known previous immunisation event, median (IQR)	
Vaccination	359 (121)
Infection	286 (417)

^a^Information on sex was based on individual self-declaration; ^b^Patients did not have a history of therapy with other immunosuppressive drugs. ^c^9 patients were assigned as previously infected based on NCAP-IgG positivity despite no known history of infection; ^c^immunisation events including vaccination and infection. Abbreviations: COVID-19, coronavirus disease 2019; IQR, interquartile range; MMF, mycophenolate mofetil; SARS-CoV-2, severe acute respiratory syndrome coronavirus 2; SD, standard deviation.

**Fig. 1:**
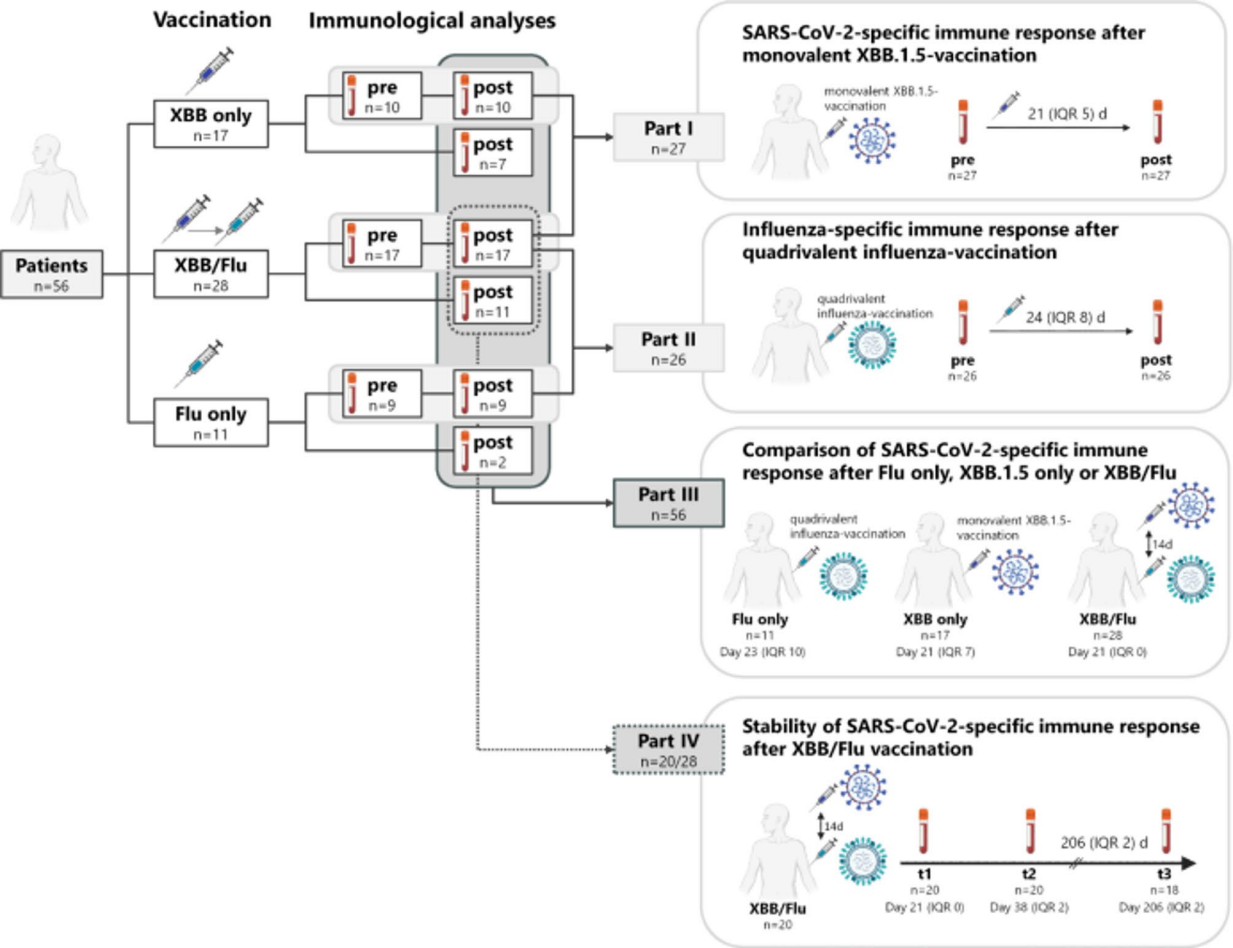
Flow-chart and schematic overview of the four parts of the study. A total of 56 patients were tested after having received either the monovalent XBB.1.5 vaccination alone (“XBB only”, *n* = 17), influenza vaccination alone (“Flu only”, *n* = 11), or sequential administration of both vaccines (“XBB/Flu”, *n* = 28, with the influenza vaccine administered fourteen days after XBB.1.5 vaccination). Among those, 36 patients had baseline analyses performed prior to vaccination (10 before XBB only, 9 before Flu only and 17 before XBB/Flu). **Part I**: SARS-CoV-2-specific humoral and cellular immune responses were characterized in 27 patients before and after monovalent XBB.1.5-vaccination irrespective of influenza vaccination. Time of testing in relation to XBB.1.5 vaccination is indicated. **Part II**: Influenza-specific humoral and cellular immune responses were characterized in 26 patients before and after influenza vaccination irrespective of XBB.1.5 vaccination. Time of testing in relation to influenza vaccination is indicated. **Part III**: SARS-CoV-2-specific immune responses were compared in dialysis patients after XBB only (*n* = 17), Flu only (*n* = 11), or sequential administration of XBB.1.5 followed by influenza vaccination (*n* = 28; XBB/Flu). Testing was performed approximately three weeks after the XBB.1.5- or influenza vaccination as indicated in the figure. **Part IV**: The stability of the SARS-CoV-2-specific immune response in 20 dialysis patients after XBB/Flu who participated in the follow-up part of the study was investigated starting at 21 days after XBB.1.5 vaccination over a period of 6 months with time points indicated in the figure. Two patients were lost to follow-up (1 died, 1 was transplanted). Demographic characteristics of the subgroups are shown in supplementary tables S1-S4.

A flow-chart and the study design are illustrated in Fig. 1. All patients were vaccinated (17 (30.4%) XBB.1.5 only, 11 (19.6%) influenza (Flu) only, and 28 (50.0%) sequential XBB.1.5/Flu), and had post-vaccination immunological analyses performed. Among those, 36 patients also had baseline analyses prior to vaccination performed (10 after XBB only, 9 after Flu only, and 17 after XBB/Flu). Immunogenicity of the monovalent XBB.1.5 and influenza vaccine was analysed in those patients before and after monovalent XBB.1.5 vaccination (Fig. 1, *n* = 27, part I), and influenza vaccination (Fig. 1, *n* = 26, part II), respectively, independent of whether patients received sequential or single administration of the individual vaccines. Comparative analysis of vaccine-induced SARS-CoV-2-specific immunity in individuals after sequential vaccination (*n* = 28), after XBB.1.5 vaccination only (*n* = 17) and after influenza vaccination (*n* = 11) was performed in part III (Fig. 1, part III). Finally, the stability of vaccine-induced SARS-CoV-2-specific immunity was analysed up to six months after sequential XBB.1.5 and influenza vaccination (Fig. 1, *n* = 20, part IV). Information on patient characteristics of the individual subgroups included in parts I-part IV is shown in supplementary tables S1-S4.

### Part I: SARS-CoV-2-specific humoral and cellular immunity after monovalent XBB.1.5 vaccination

**Fig. 2:**
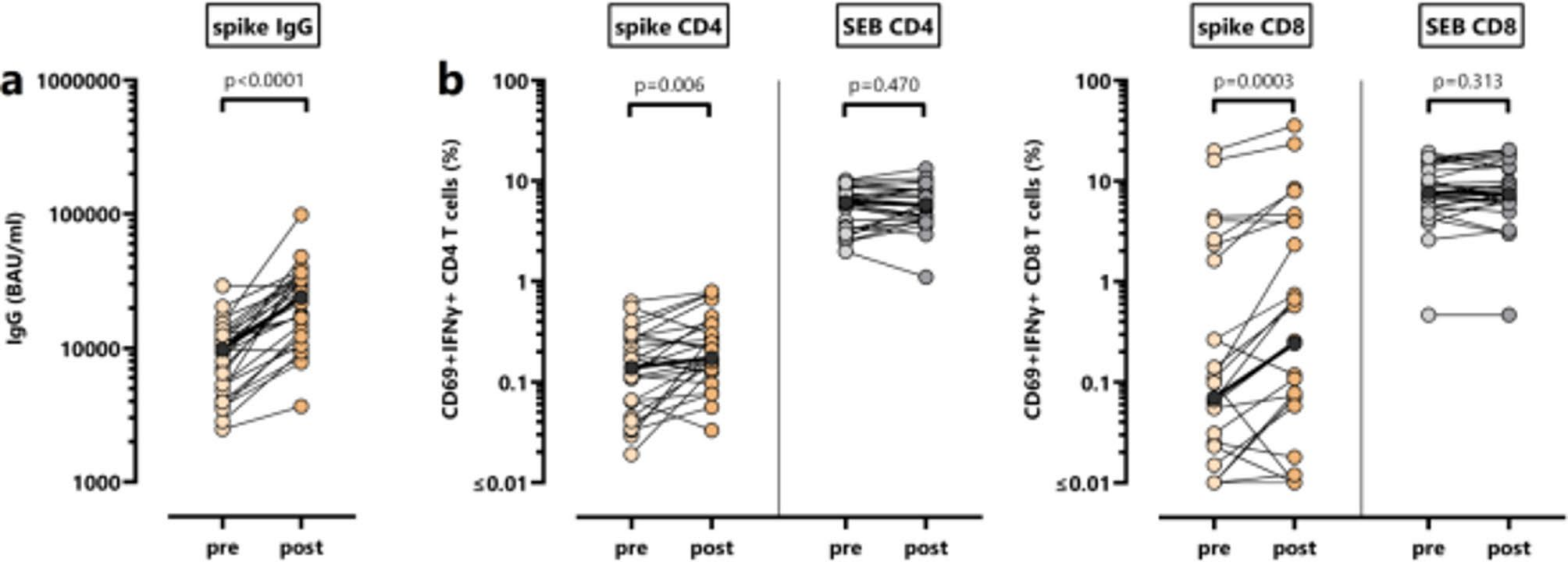
Vaccine-induced SARS-CoV-2-specific humoral and cellular immune response in dialysis patients after monovalent XBB.1.5 vaccination. Blood samples were drawn from dialysis patients (*n* = 27) before and a median of 21 (IQR 5) days after monovalent XBB.1.5 vaccination. Levels of spike-specific **(a)** IgG antibodies towards parental spike protein, as well as spike-specific and SEB-reactive **(b)** CD4 and CD8 T cells were analysed. Highlighted symbols represent medians. Differences were calculated using the Wilcoxon signed rank test. Abbreviations: BAU, binding antibody unit; IFN, interferon; Ig, immunoglobulin; IQR, interquartile range; SEB, *Staphylococcus aureus* Enterotoxin B.

SARS-CoV-2-specific humoral and cellular immune responses after XBB.1.5 vaccination was analysed from paired samples of 27 patients immediately before and a median of 21 (IQR 5) days after vaccination (Fig. 1, part I; Fig. 2). Information on patient characteristics is shown in supplementary table S1. All patients had spike-specific IgG antibody levels above detection limit prior to vaccination (median 9743 (IQR 7983) BAU/ml, which increased to 23907 (IQR 21403) BAU/ml thereafter (*p* < 0.0001, Fig. 2a). Spike-specific T cells were characterized after stimulation with overlapping peptides spanning the parental spike protein and quantified flow-cytometrically based on co-expression of the activation marker CD69 and the cytokine IFNγ. In line with IgG antibodies, spike-specific CD4 and CD8 T cells increased significantly after vaccination (*p* = 0.006 and *p* = 0.0003, respectively, Fig. 2b), although spike-specific CD8 T cells showed higher interindividual variability. Differences were spike-specific, as SEB-reactive CD4 and CD8 T-cell levels did not change after vaccination (Figs. 2b). We found an inverse correlation between pre-existing spike-specific IgG and CD4 T-cell levels and the respective fold increase after vaccination (*p* = 0.043, Spearman *r*=-0.393 for IgG; *p* = 0.02, *r*=-0.416 for CD4 T cells), whereas no such correlation was observed for CD8 T cells (*p* = 0.181, *r* = 0.266).

To analyse potential differences in CD4 and CD8 T-cell levels toward spike from the parental strain and Omicron variant XBB.1.5, blood samples from a subset of patients after vaccination were also stimulated with peptide pools derived from XBB.1.5 spike. As shown in supplementary figure S1, the percentages of XBB.1.5-spike specific CD4 and CD8 T-cell levels did not differ from the respective T-cell frequencies against parental spike, indicating substantial T-cell cross reactivity between the strains.

**Fig. 3:**
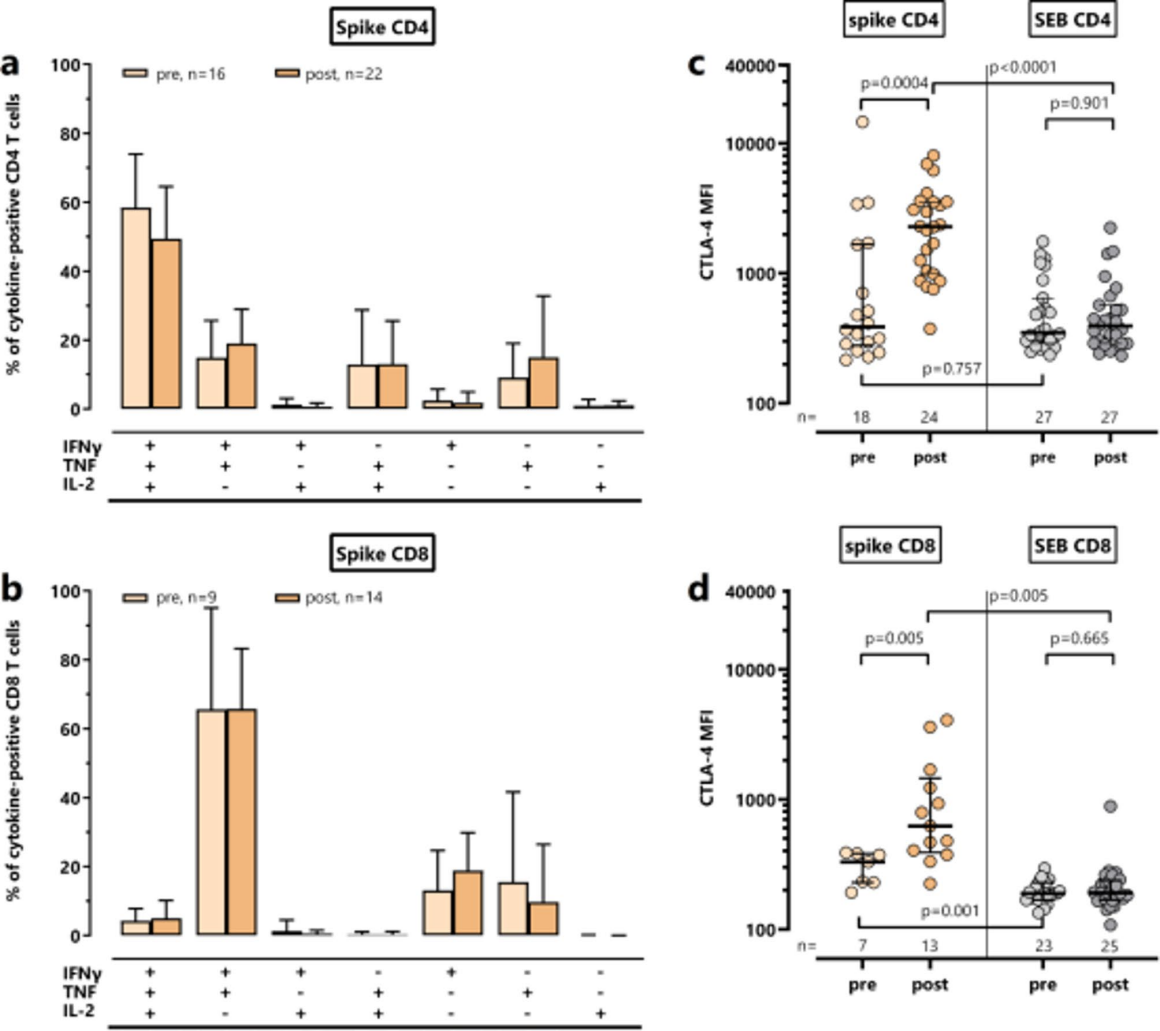
Functional and phenotypical characterization of parental spike-specific CD4 and CD8 T cells in dialysis patients before and after monovalent XBB.1.5 vaccination. Cytokine expression profiles of **(a)** CD4 and **(b)** CD8 T cells after stimulation with parental spike protein or *Staphylococcus aureus* Enterotoxin B (SEB) were compared in patients before and after XBB.1.5 vaccination. At the single-cell level, the cytokine-expressing T cells were differentiated into 7 subpopulations according to their expression of IFNγ, TNF and IL-2 (single, double or triple cytokine-expressing cells). All samples were analysed but only samples of patients with at least 30 cytokine-expressing CD4 and CD8 T cells were included, respectively, to allow for robust statistical analysis (datasets indicated in the figures). Bars represent means and standard deviations of subpopulations. Differences were determined using the unpaired t-test. Median fluorescence intensity (MFI) of CTLA-4 expression on spike-specific and SEB-reactive **(c)** CD4 and **(d)** CD8 T cells was compared. All samples were analysed, but to allow robust statistical analysis, only samples with at least 20 cytokine-positive CD4 and CD8 T cells, respectively, were included (datasets indicated in the figures). Horizontal bars refer to the median values with interquartile range. Differences between groups were analysed using the Mann Whitney test. Differences remain significant if only paired datasets are considered. Abbreviations: CTLA-4, cytotoxic T-lymphocyte-associated protein 4; IFN, interferon; IL, interleukin; RG, reference group; SEB, *Staphylococcus aureus* Enterotoxin B; TNF, tumor necrosis factor.

Further phenotypical and functional characteristics of spike-specific T cells were analysed by co-expression of IFNγ, IL-2 and TNF after Boolean gating. This allowed distinction of seven subpopulations including polyfunctional cells simultaneously expressing all three cytokines, two cytokines, or one cytokine only. Both before and after vaccination, spike-specific CD4 T cells were predominantly polyfunctional, followed by CD4 T cells expressing TNF alone or in combination with IFNγ or IL-2 (Fig. 3a). Cytokine profiles of spike-specific CD4 T cells differed from those of spike-specific CD8 T cells. As expected, CD8 T cells produced less IL-2, and were primarily IFNγ^+^TNF^+^ (Fig. 3b). Furthermore, expression levels of CTLA-4 as an immunological regulatory marker and as an indicator of a recent antigen exposure were analyzed (Fig. 3c and d). Median CTLA-4 expression levels showed a significant increase on both spike-specific CD4 (*p* = 0.0004) and CD8 T cells (*p* = 0.005) after vaccination. Likewise, CTLA-4 expression on both vaccine-induced spike-specific CD4 T cells and CD8 T cells were significantly higher compared to SEB-reactive T cells (Fig. 3c and d). Of note, despite low sample size, even baseline levels of CTLA-4 were higher on spike-reactive CD8 T cells than on respective SEB-reactive T cells which may result from a more recent immunological challenge with SARS-CoV-2 antigens.

### Part II: Influenza-specific humoral and cellular immunity after quadrivalent influenza vaccination

**Fig. 4:**
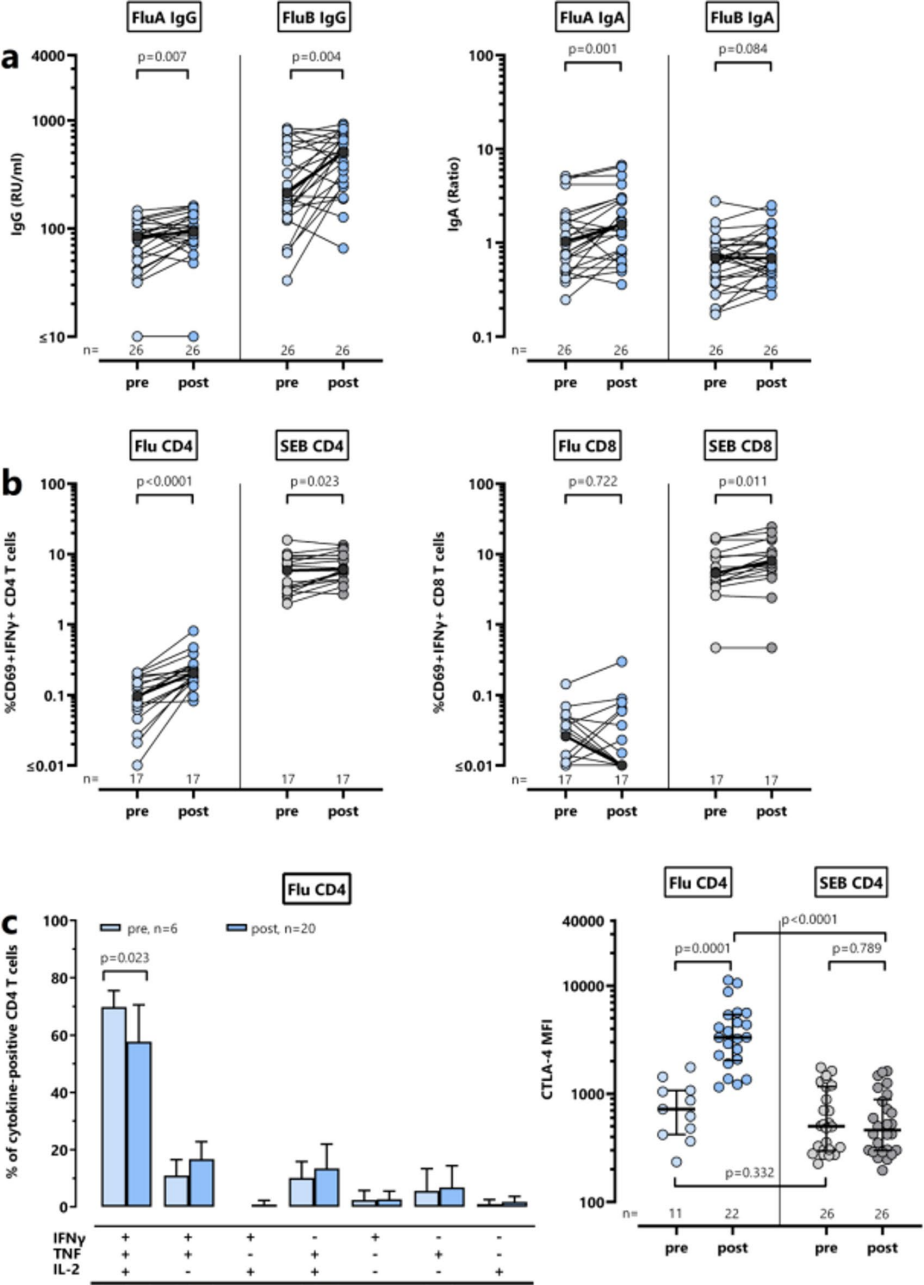
Vaccine-induced Influenza-specific humoral and cellular immunity in dialysis patients before and after quadrivalent influenza vaccination. Blood samples were drawn from dialysis patients (*n* = 26) before and a median of 24 (IQR) 8 days after influenza vaccination (*n* = 21 Influsplit Tetra, *n* = 5 Efluelda). Levels of specific **(a)** IgG and IgA antibodies towards influenza A and B, as well as **(b)** influenza-specific and SEB-reactive CD4 and CD8 T cells were analysed. Not all patients had pre-vaccination T-cell analyses performed with tested individuals indicated in the figures. Highlighted symbols represent medians. Differences were calculated using the the Wilcoxon signed rank test. Stratified analyses of data between patients who had received Influsplit and Efluelda are shown in supplementary figure S2. **(c)** Cytokine expression profiles of influenza-specific CD4 T cells were compared before and after vaccination. At the single-cell level, the cytokine-expressing T cells were differentiated into 7 subpopulations according to their expression of IFNγ, TNF and IL-2 (single, double or triple cytokine-expressing cells). All samples were analysed but only samples of patients with at least 30 cytokine-expressing CD4 and CD8 T cells were included, respectively, to allow for robust statistical analysis (datasets indicated in the figures). Bars represent means and standard deviations of subpopulations. Differences were determined using the unpaired t-test. **(d)** Median fluorescence intensity (MFI) of CTLA-4 expression on influenza-specific and SEB-reactive CD4 T cells was compared. All samples were analysed, but to allow robust statistical analysis, only samples with at least 20 cytokine-positive CD4 and CD8 T cells, respectively, were included (datasets indicated in the figures). Horizontal bars refer to the median values with interquartile range. Differences between groups were analysed using the Mann Whitney test. Differences remain significant if only paired datasets are considered. Abbreviations: CTLA-4, cytotoxic T-lymphocyte-associated protein 4; IFN, interferon; IL, interleukin; RG, reference group; SEB, *Staphylococcus aureus* Enterotoxin B; TNF, tumor necrosis factor.

The induction of influenza-specific humoral and cellular immune response after influenza vaccination was analysed from paired samples for twenty-six patients immediately before and a median of 24 (IQR 8) days after vaccination (Fig. 1, part II; Fig. 4). Information on patient characteristics of this subgroup is shown in supplementary table S2. Antibody titers directed against the two influenza A and the two influenza B components of the vaccine were examined separately. Levels of IgG antibodies toward influenza A (*p* = 0.007) and B (*p* = 0.004) significantly increased after vaccination (Fig. 4a). Levels of IgA antibodies toward influenza A also increased (*p* = 0.001), whereas the increase in IgA levels towards influenza B did not reach statistical significance (*p* = 0.084, Fig. 4a). Influenza-specific T cells were characterized after stimulation with the tetravalent influenza vaccine. Influenza-specific CD4 T-cell levels showed a significant increase (*p* < 0.0001, Fig. 4b). In contrast, influenza-specific CD8 T-cell levels were not induced after vaccination (*p* = 0.722, Fig. 4b). We do not have any evidence for differences in the two different quadrivalent vaccines used (supplementary figure S2). Finally, SEB-reactive CD4 or CD8 T-cell levels did not change after vaccination (Fig. 4b).

Functional analysis showed that influenza-specific CD4 T cells were mostly polyfunctional, followed by double-positive cells co-expressing either TNF/IFNγ or TNF/IL-2 (Fig. 4c). Similar as with spike-specific CD4 T-cell characteristics, the cytokine profile after vaccination showed a decrease in polyfunctional T cells with a concomitant shift toward higher expression of double-cytokine producing populations. Again, expression of CTLA-4 on influenza-specific CD4 T cells increased significantly after vaccination (Fig. 4d), and reached higher levels than respective SEB-reactive CD4 T cells. In contrast, baseline levels of CTLA-4 on influenza-reactive CD4 T cells were similar as on SEB-reactive T cells.

### Part III: Influence of a sequential influenza vaccination on XBB.1.5-vaccine-induced SARS-CoV-2-specific humoral and cellular immune responses

**Fig. 5:**
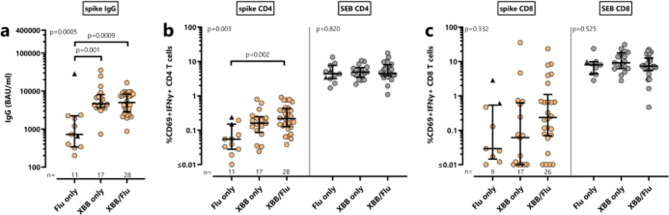
Comparison of vaccine-induced spike-specific humoral and cellular immunity in dialysis patients after sequential, monovalent XBB.1.5 alone or quadrivalent influenza vaccination alone. Blood samples were drawn from dialysis patients at a median of 23 (IQR 10) days after quadrivalent influenza vaccination (Flu only, *n* = 11), at a median of 21 (IQR 7) days after monovalent XBB.1.5 vaccination alone (XBB only, *n* = 17) or at a median of 21 (IQR 0) days after sequential XBB1.5 + Flu vaccination (XBB/Flu, *n* = 28, quadrivalent influenza vaccine was administered fourteen days later than XBB.1.5 vaccine). Levels of **(a)** spike-specific IgG antibodies, as well as **(b)** CD4 and **(c)** CD8 T cells after stimulation with peptides toward the parental spike protein or *Staphylococcus aureus* enterotoxin B (SEB) were compared between the three groups. Two patients, marked by a black triangle, had a previous SARS-CoV-2 infection 20 and 56 days before administration of the influenza vaccine, respectively. Bars represent medians with interquartile ranges. Differences between groups were analysed using Kruskal-Wallis test followed by Dunn’s post test. Abbreviations: BAU, binding antibody unit; Flu, influenza, IFN, interferon; Ig, immunoglobulin.

A potential influence of a sequential influenza vaccination on the XBB.1.5-induced immune response was investigated by comparing spike-specific immunity of 28 patients who had analyses performed after sequential vaccination (XBB/Flu) with respective data of 17 patients receiving XBB.1.5 vaccine alone (“XBB only”) and of 11 patients receiving influenza vaccine alone (“Flu only”, Fig. 1, part III; Fig. 5). Information on patient characteristics of these subgroups are shown in supplementary table S3. Despite sequential influenza vaccination after XBB.1.5, patients reached median levels of spike-specific IgG of 4981 BAU/ml (IQR 5521 BAU/ml) of similar magnitude as in patients after XBB.1.5 only (4641 BAU/ml (IQR 4407 BAU/ml)), which expectedly were significantly higher than in patients after Flu only vaccination (726 BAU/ml (IQR 1897 BAU/ml), *p* = 0.0005, Fig. 5a). As with IgG, spike-specific CD4 T-cell levels showed significant differences between the groups (*p* = 0.003), and were highest in patients receiving the XBB.1.5 vaccine followed by the influenza vaccine (*p* = 0.002, Fig. 5b), with no significant difference between patients after XBB.1.5 vaccination alone (0.16% (IQR 0.16%)) and sequential vaccination (0.22% (IQR 0.30%), *p* = 0.659). Despite some trend toward numerically highest levels of spike-specific CD8 T cells in the XBB/Flu vaccine group, the differences between the groups did not reach statistical significance (*p* = 0.332, Fig. 5c). Differences in CD4 T cells were spike-specific, as global T-cell activation capacity after SEB-stimulation were similar in the three groups (Fig. 5b and c). Baseline levels of specific antibodies and T cells tested prior to vaccination did not differ between the three groups, although there was a trend towards lower values in the Flu only group (supplementary figure S3).

It is interesting to note that the highest spike-specific CD4 and CD8 T-cell levels in the Flu only group were observed in two patients with a history of recent SARS-CoV-2 infection (20 and 56 days before administration of the influenza vaccine, respectively, marked by a triangle, Fig. 4). Their median CD4 and CD8 T-cell levels were in a similar or even higher range as with those of the XBB.1.5 vaccine groups. The IgG titers of these two patients differed, with the higher titer observed in the patient with the shorter time from infection (28431.54 BAU/ml vs. 653.95 BAU/ml).

We finally applied multivariable linear regression analysis adjusted for age, sex, and prior infection, which confirmed differences in vaccine-induced IgG and CD4 T-cell levels between the three regimens, with lowest levels in patients after Flu only vaccination (supplementary table S5). Moreover, IgG were also confounded by sex, whereas prior infection had no confounding effect on any of the parameter.

### Part IV: Stability of spike-specific immune responses after sequential XBB.1.5 + Flu vaccination

**Fig. 6:**
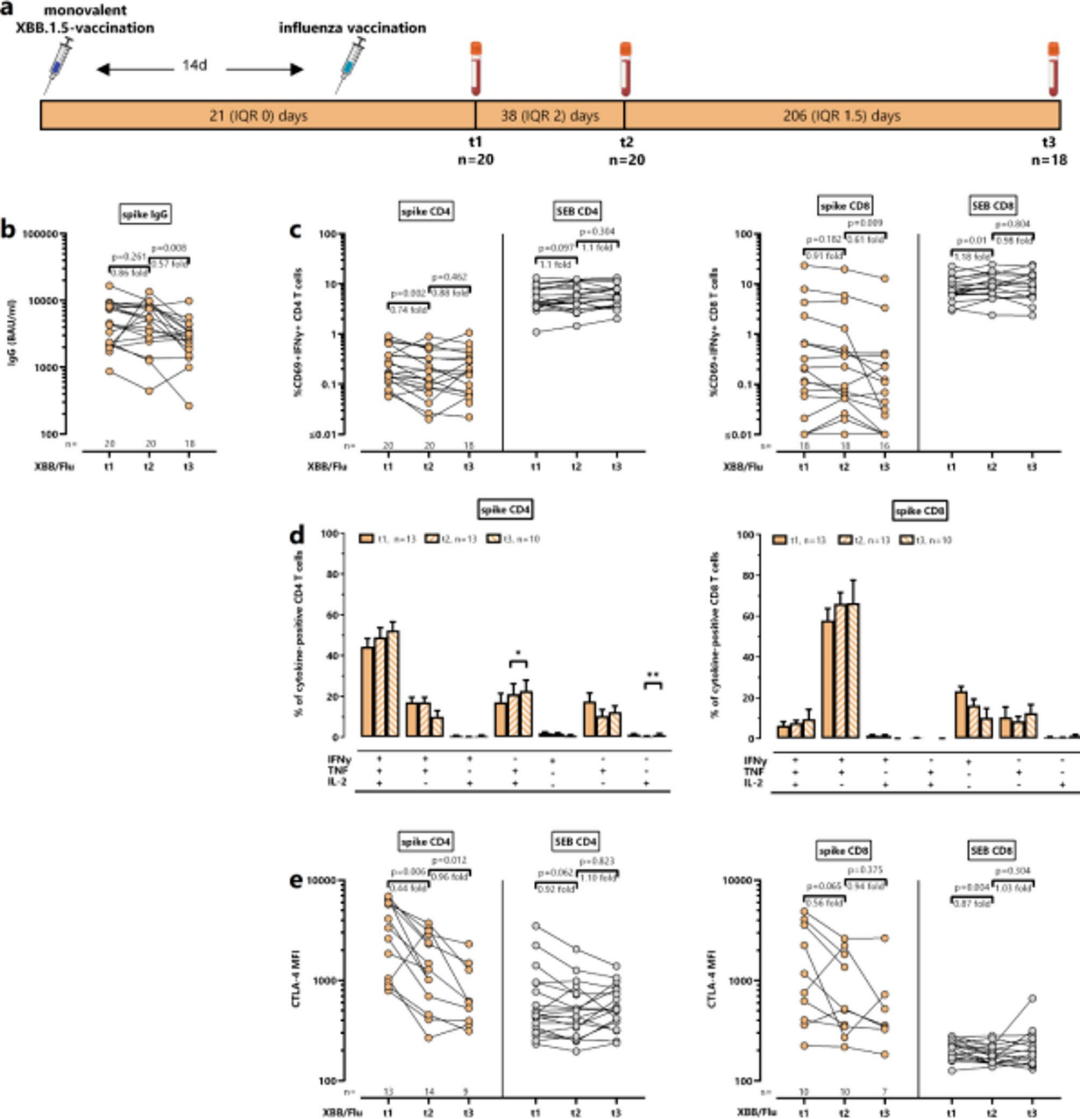
Stability of spike-specific immune responses after sequential XBB.1.5 + Flu vaccination over six months. **(a)** Schematic outline of the study design. Blood samples were drawn from a subgroup of 20 dialysis patients receiving the monovalent XBB.1.5 vaccine followed by the quadrivalent influenza vaccine fourteen days later (XBB/Flu), who consented to participate in the follow-up part of the study. Time points (t1-t3) for blood sampling refer to the XBB.1.5 vaccination, starting 21 (IQR 0) days after vaccination. During the 6-month period, one female patient in her late seventies died (secondary chronic renal disease, 6 years on dialysis), and one male patient in his sixties received a transplant (secondary chronic renal disease, 2.4 years on dialysis) 105 and 91 days after XBB.1.5 and influenza vaccination, respectively. Levels of **(b)** spike-specific IgG antibodies, **(c)** CD4 and CD8 T cells after stimulation with peptides toward the parental spike protein or *Staphylococcus aureus* enterotoxin B (SEB) were compared between the three different time points **(d)** Cytokine-expression profiles of CD4 and CD8 T cells after stimulation with parental spike protein or SEB were compared between the three different time points. Only samples of the patients with at least 30 cytokine-expressing CD4 or CD8 T cells were included, respectively, to allow for robust statistical analysis (datasets indicated in the figures). **(e)** Median fluorescence intensity (MFI) of CTLA-4 expression on spike-specific and SEB-reactive CD4 and CD8 T cells from patients after sequential vaccination was determined. To allow robust statistical analysis, only samples with at least 20 cytokine-positive CD4 and CD8 T cells, respectively, were included. Bars represent (b, c, e) medians with interquartile ranges or (d) means and standard deviations for cytokine-subpopulations. Fold changes are indicated above the graphs and were calculated by dividing the individual levels after vaccination and levels prior to vaccination. Paired analysis between time points were analysed using Wilcoxon-signed rank test. Abbreviations: CTLA-4, cytotoxic T-lymphocyte-associated protein 4; IFN, interferon; Ig, immunoglobulin; IL, interleukin; IQR, interquartile range; SEB, *Staphylococcus aureus* Enterotoxin B; TNF, tumor necrosis factor.

Starting from 21 (IQR 0) days after XBB.1.5 vaccination, we finally analysed the stability of the vaccine-induced spike-specific immune response two times over a 6-month time period among 20/28 patients who had received sequential XBB.1.5 + Flu vaccination (t1/t2, *n* = 20; t3, *n* = 18, Fig. 1, part IV; Fig. 6a). Information on patient characteristics of these subgroups are shown in supplementary table S4.

Compared to vaccine-induced immunity after 21 days (t1), spike-specific IgG levels decreased 0.86-fold by day 38. This level further decreased 0.57-fold and reached statistical significance by 6 months after vaccination (*p* = 0.008, Fig. 6b). Likewise, median percentages of spike-specific CD4 T cells showed a significant decrease after 38 days (t2, 0.74-fold, *p* = 0.002), but remained largely stable subsequently (*p* = 0.462, Fig. 6c). Spike-specific CD8 T-cell levels remained rather stable early after vaccination (t2, 0.91-fold, *p* = 0.182), and decreased significantly by 6 months (t3, 0.61-fold, *p* = 0.009, Fig. 6c). In general, no pronounced dynamic changes were observed among SEB-reactive CD4 or CD8 T-cell frequencies over time. Regarding functional and phenotypical characteristics, the overall cytokine expression profiles of spike-specific CD4 and CD8 T cells largely remained stable with some trend in increasing polyfunctionality over time (Fig. 6d). As expected, CTLA-4 expression on spike-specific CD4 and CD8 T cells was numerically highest early after vaccination, and decreased over the 6-month period from a median of 3345 (IQR 5070) to 604 (IQR 1005) on CD4 T cells, and from 963.5 (IQR 3312.7) to 516 (IQR 1596) on CD8 T cells after 38 days post-vaccination, with no further decrease by 6 months (0.94-fold, *p* = 0.375, Fig. 6e). Despite some decrease in spike-specific immunity over a period of six months, levels of IgG antibodies and CD4 T cells remained significantly higher in patients receiving the XBB.1.5 vaccine as compared to a subgroup of 23 patients without XBB.1.5 vaccination, who were recruited in the same time period (Table 2). An exception were spike-specific CD8 T cells which did not differ between the two groups (Table 2).

**Table 2:** Spike-specific immune responses six months after sequential XBB/Flu vaccination compared to patients without XBB.1.5 vaccination.

Follow-up testing	XBB/Flu *n* = 18	No XBB.1.5 vaccination *n* = 23	*p* value
Spike-specific IgG antibodies (BAU/ml), median (IQR)	2998 (1990)	1155 (2931.9)	0.033
Spike-specific CD4 T cells (% CD69^+^IFNγ^+^), median (IQR)	0.17 (0.29)	0.08 (0.07)	0.021
Spike-specific CD8 T cells (% CD69^+^IFNγ^+^), median (IQR)	0.09 (0.33) *n* = 16	0.03 (0.05)	0.148

Mann-Whitney Test; during the 6-month study period, no COVID-19 infections were reported; abbreviations: BAU, binding antibody unit; IFN, Interferon; Ig, immunoglobulin; IQR, interquartile range. Patients who were not vaccinated (mean age 68.6 ± 15.8 years; 13 males, 10 females) were recruited at the same time as the XBB/Flu-vaccinated patients.

## Discussion

Annual vaccination towards COVID-19 and influenza is now becoming standard of care before the seasonal waves, and the vaccines can either be co-administered on the same day or sequentially on separate days. End-stage renal disease and intermittent dialysis treatment are risk factors for severe COVID-19 and influenza disease, although knowledge of immunogenicity and the effects of consecutive vaccination series on cellular and humoral spike-specific immunity in dialysis patients is limited. We now show that both the monovalent XBB.1.5 vaccine and a quadrivalent influenza vaccine strongly induced pre-existing humoral and cellular immunity toward the respective antigens in dialysis patients. The immune responses were considerably stable over time, and we did not find any evidence that the XBB.1.5-induced immune response was adversely affected by a subsequent influenza vaccination.

Baseline analysis prior to vaccination showed that the majority of dialysis patients exhibited a SARS-CoV-2-specific immunity resulting from at least three prior antigen exposures including vaccinations and/or infections. Evidence for continued exposure with the virus was given by the fact that hybrid immunity based on a known history of infection or NCAP-specific immunity was found in more than 75% of patients, which represents a relative increase of at least 18% compared to dialysis patients recruited a year before from the same dialysis center^7^. As with spike-specific immunity, dialysis patients exhibited detectable influenza-specific antibodies and T cells at baseline, in line with regular exposure and annual vaccine recommendations^17,18^. Unlike insufficient de novo induction of immune responses in immunologically naïve patients^19–21^, we show that both the XBB.1.5 and the influenza vaccine strongly boosted pre-existing humoral and cellular immunity in dialysis patients. Regarding cellular immunity, the SARS-CoV-2-mRNA vaccine elicited both CD4 and CD8 T cells, whereas the protein-based influenza vaccine primarily induced CD4 T cells. Both spike- and influenza-specific CD4 T cells were mainly polyfunctional, with some functional and phenotypical alterations in the early induction phase after vaccination. Early after vaccination, both vaccines led to a relative decrease in multifunctionality and an increase in CTLA-4 expression on the respective specific T-cell population, which reflect immunological exposure to the respective vaccine antigens and also potential regulatory mechanisms limiting excessive responses^22^. These dynamics represent typical physiological features associated with the expansion of Th_1_ cells following SARS-CoV-2^11,23–25^, and influenza vaccination^6,23,24^. Although the monovalent XBB.1.5 vaccine encodes only the spike protein of the Omicron subvariant XBB.1.5, vaccine-induced spike-specific CD4 and CD8 T cells showed pronounced cross-reactivity between XBB.1.5 and the parental SARS-CoV-2 strain. This cross-reactivity with XBB.1.5 was also found in immunocompetent individuals^25^, and with previously circulating variants such as BA.4/5 in both patients^7^ and controls^26^. Spike-specific antibodies were also strongly induced^7,26^, although cross-reactivity at the level of neutralizing activity is generally less pronounced. However, other studies have reported potent XBB.1.5-vaccine induced IgG antibodies with strong correlation with neutralizing activity in both immunocompetent individuals^25–29^ and hemodialysis patients^8^,

Overall, we have shown that both vaccines lead to a strong immune response in dialysis patients. However, given that mRNA vaccines are still in their early development, knowledge on the translation of vaccine-antigen and immunogenicity when combined with conventional vaccines is still limited. Based on as yet poorly characterized mechanisms including trained immunity, a vaccine such as influenza^30^ or BCG^31^ may affect immunity and/or susceptibility toward infections other than the pathogen targeted by the vaccine^32^. Examples may also include herpes zoster episodes after vaccination with COVID-19 vaccines^33^ or with the inactivated shingles vaccine^34^, which both are known to have strong adjuvant activity^35–37^. A similar mechanism may also apply to immune responses upon sequential vaccinations, and may in part depend on the strength of the adjuvant effect of one vaccine that could affect induction of an immune response by the subsequent vaccine. However, results from the TACTIC study in immunocompetent individuals over 60 years of age^12^ did not show any significant differences in humoral immunity between groups receiving the COVID-19 vaccine alone, the COVID-19 vaccine followed by influenza vaccination, or the reverse vaccination sequence. These findings argue against a relevant immunological interference between an mRNA-based COVID-19 booster and a quadrivalent influenza vaccine. Likewise, our study did not provide any evidence that the quantity of XBB.1.5 vaccine-induced antibody and T-cell responses in dialysis patients were altered by subsequent influenza vaccination. Furthermore, administration of the quadrivalent influenza vaccine alone did not non-specifically induce any spike-specific humoral or cellular immune response. This lack of mutual interference is further supported by the fact that phenotypical and functional changes early after vaccination were confined to the respective T cells targeted by the vaccine and were not observed in polyclonal T-cell populations of other specificities. Despite the absence of correlates of protection, these immunological data suggest that both vaccines can be administered without any evidence of compromising each other’s efficacy. These data are encouraging in light of the ongoing development of new mRNA-based vaccines toward other pathogens that may require administration in close temporal proximity to influenza vaccines.

Previous studies have described a reduced and less durable immune response following COVID-19 primary and booster immunisations in dialysis patients compared with immunocompetent individuals^38–40^. However, repeated vaccinations and natural infections contribute to the continuous maturation of immunological memory, leading to more efficient reactivation of B- and T-memory cells^41–43^. This is supported by our observation that the immune response stabilized at a level higher than non-vaccinated patients tested in the same time period. Although this prolonged stability of the immune response may result from repeated exposure to SARS-CoV-2 antigens through intermittent infections, patients did not report any COVID-19 infections during our observation period. Thus, the observed stability underscores the benefit of booster vaccinations even in individuals with impaired immune function.

A strength of our study was the first-time investigation of potential immunological interference between sequential administration of the monovalent XBB.1.5 booster and the subsequent quadrivalent influenza vaccine in dialysis patients. Moreover, patients were vaccinated during the same time period and in the same region where similar variants circulated before and after vaccination. A limitation is the restriction of antibody testing to IgG without data on neutralizing activity towards the vaccine strains. However, we have performed a detailed quantification and characterization of the cellular immune responses including stability of the booster response. A further limitation is the real-world observational setting with convenience sampling of in part small subgroups where the timing of post-vaccine sampling was primarily guided by the three routine vaccination regimens applied in the dialysis unit (i.e. either COVID-19 or influenza vaccine alone or COVID-19 followed by influenza). Moreover, a small number of dialysis patients received the high-dosed quadrivalent influenza vaccine, but we did not find any evidence for any difference in immune responses between patients receiving standard-dose and high-dose vaccination.

In conclusion, sequential administration of the COVID-19 and influenza vaccines was not associated with compromised immunogenicity of either vaccine, and is a practically feasible regimen for dialysis units, where patients return on a regular basis. Unlike co-administration on the same day in two different arms, a sequential series with the use of the ipsilateral side for each vaccine is also favourable to avoid the shunt arm. We showed that the monovalent XBB.1.5 and quadrivalent influenza vaccines led to a strong induction of pre-existing baseline immunity, which is of clinical relevance regarding annual vaccine recommendations toward these respiratory pathogens. Similarly, a pronounced booster effect on specific antibodies and T cells was recently demonstrated for the newly approved mRNA- and protein-based vaccines towards the respiratory syncytial virus (RSV) in both transplant recipients^44–46^ and patients with chronic kidney disease^47^. At present, the stability of RSV-vaccine induced immunity is unknown. RSV vaccination is currently recommended once prior to the RSV infection season but may be integrated in a sequential series of respiratory seasonal vaccines. Overall, our data provide important insights for future vaccination strategies and may help refine recommendations to optimize seasonal vaccinations in immunocompromised patients.

## Methods

### Study design and subjects

In this real-world observational study, patients undergoing hemodialysis or continuous ambulatory peritoneal dialysis, and who were willing to participate were enrolled at the SHG Clinic in Völklingen, Germany from July to October 2023. A subgroup of patients was analysed for pre-existing SARS-CoV-2- and influenza-specific humoral and cellular immunity. Enrolment was restricted to patients without vaccination toward influenza and COVID-19 in the last three months, and without clinical evidence of concurrent SARS-CoV-2, influenza or other respiratory infections. All patients receiving sequential vaccinations of Comirnaty Omicron XBB.1.5 (BioNTech/Pfizer), followed by the quadrivalent influenza vaccine (including A/Victoria/4897/2022 (H1N1)pdm09, A/Darwin/9/2021 (H3N2), B/Austria/1359417/2021, B/Phuket/3073/2013, either standard-dose Influsplit Tetra (GSK) or high-dose Efluelda (Sanofi)) fourteen days later were consecutively enrolled from November 2023 to May 2024. Moreover, patients who chose only one vaccine on a voluntary basis (either the XBB.1.5 vaccine or the influenza vaccine only) were recruited as control groups. Heparinized blood samples for analyses of SARS-CoV-2-and/or influenza-specific humoral and cellular immunity were collected before and at defined time points after vaccination (Fig. 1). Vaccinations were given intramuscularly in the arm contralateral to the shunt after the dialysis procedure, and blood samples were collected prior to dialysis on the day of the dialysis session. All patients received a questionnaire for self-reporting their history of COVID-19 vaccination and infection. In addition, NCAP-IgG ELISA was performed to independently assess evidence of infection in patients without known history of COVID-19 infection. The study was performed in adherence to the declaration of Helsinki and approved by the ethics committee of the Ärztekammer des Saarlandes (reference 76/20 including amendments), and written informed consent was obtained from all patients.

### Quantitative, phenotypical and functional analysis of spike- and influenza-specific T-cell responses

To determine spike- and influenza-specific T cells, a 6 h-stimulation of heparinized whole blood samples was performed as previously described^23,48^. In brief, samples were stimulated in the presence of co-stimulatory antibodies against CD28 and CD49d (clone L293 and clone 9F10, 1 µg/ml each) with overlapping peptides (each peptide 2 µg/ml) spanning the parental spike or the Omicron variant XBB.1.5-spike protein (jpt Berlin, Germany), titrated amounts of the quadrivalent influenza vaccine (8 µl/225µl blood; GlaxoSmithKline), 0.64% DMSO (negative control) and 2.5 µg/ml of *Staphylococcus aureus* enterotoxin B (SEB, positive control; Sigma), respectively. Brefeldin A (10 µg/ml) was added at 2 h. After another 4 h of stimulation, samples were treated with 20mM EDTA for 15 min and cells were fixed using BD lysing solution. After fixation, leukocytes were permeabilized with 2 ml of FACS buffer containing 0.1% saponin (Sigma) for 10 min; thereafter, cells were immunostained using anti-CD4 (clone SK3, 1:33.3), anti-CD8 (clone SK1, 1:12.5), anti-CD69 (clone L78, 1:33.3), anti-IFNγ (clone 4 S.B3, 1:100), anti-IL-2 (clone MQ1-17H12, 1:12.5), anti-TNF (clone MAb11, 1:20), and anti-CTLA-4 (clone BNI3, 1:50) and analysed using flow cytometry (BD FACS Canto II and FACSDiva software 6.1.3.). Based on previous studies^48^–^24,26^, activation based on CD69-positivity and IFNγ-induction after stimulation were used as highly specific markers to identify spike- and influenza-reactive CD4 or CD8 T cells. Quantification of specific CD4 and CD8 T-cell levels was performed by subtraction of respective levels after control stimulation with detection limits of 0.03% for specific CD4 T cells and 0.06% for specific CD8 T cells as established previously^26^. T-cell functionality was further characterized by analyzing co-expression of IL-2 and/or TNF in cells with and without IFNγ-expression, as well as the cytotoxic T-lymphocyte-associated protein 4 (CTLA-4). Polyfunctionality was defined as co-expression of all three cytokines, as compared to two cytokines, or one cytokine only. The gating strategy is shown in supplementary figure S4.

### Determination of SARS-CoV-2- and influenza-specific antibodies

Antibody tests were performed according to the manufacturer’s instructions (Euroimmun, Lübeck, Germany) as previously described^23,48^. SARS-CoV-2-specific IgG antibodies toward the receptor binding domain of the parental SARS-CoV-2-spike protein were quantified using the enzyme-linked immunosorbent assay (ELISA, SARS-CoV-2-QuantiVac). Antibody binding units (BAU/ml) < 25.6 were scored negative, ≥ 25.6 and < 35.2 were scored intermediate, and ≥ 35.2 were scored positive. Anti-SARS-CoV-2-NCP-ELISA was used to determine SARS-CoV-2-specific IgG toward the nucleocapsid (NCAP) protein. NCAP-ELISA positivity served as independent evidence for infection in patients without known COVID-19 infection history.

Influenza-specific IgA and IgG antibodies were determined using Anti-Influenza-A/B-Virus-ELISA. IgA levels represent semiquantitative values calculated by dividing the ratio between the extinction of the serum sample and the extinction of a calibration serum corresponding to the upper limit of the normal range. Ratios < 0.8 were scored negative, ≥ 0.8 and < 1.1 were scored intermediate, and ≥ 1.1 were scored positive. IgG levels expressed in relative units (RU/ml) were scored as negative for values < 16, intermediate for values ≥ 16 and < 22 and positive for values ≥ 22.

### Statistical analysis

Statistical analyses were carried out using GraphPad Prism 10.6.0 software (GraphPad, San Diego, CA, USA) using two-tailed tests. Unpaired nonparametric data between groups were compared using Mann-Whitney test or Kruskal-Wallis test followed by Dunn’s multiple comparison test. Wilcoxon matched pairs test was used to compare paired data between two groups. Correlations between pre-existing immunity and increase after vaccination were calculated using Spearman’s rank testing. Multivariable linear regression analysis was performed to test the effects of age, sex, prior infection and vaccine regimen on spike-specific IgG and CD4 and CD8 T cells. In case datasets were missing, this is specified in each figure or table legend. The fold change was calculated as a ratio between post- and pre-vaccination values; to avoid division by 0 and overestimation of fold changes with low pre-vaccination levels, a pseudocount-based on the assay detection limit of 0.03% and 0.06% was added to each percentage of CD4 and CD8 T cells prior to division, respectively. A p value < 0.05 was considered statistically significant.

## Supplementary Information

Below is the link to the electronic supplementary material.

Supplementary Material 1

## Data Availability

All figures and tables have associated raw data. The data that support the findings of this study are available from the corresponding author upon request.

## References

[1] Wat, D. The common cold: a review of the literature. *Eur. J. Intern. Med.* **15**, 79–88 (2004). 15172021 10.1016/j.ejim.2004.01.006 PMC7125703

[2] Eccles, R. Understanding the symptoms of the common cold and influenza. *Lancet Infect. Dis.* **5**, 718–725 (2005). 16253889 10.1016/S1473-3099(05)70270-X PMC7185637

[3] Pavia, A. T. Viral infections of the lower respiratory tract: old viruses, new viruses, and the role of diagnosis. *Clin. Infect. Dis.* **52** (Suppl 4), S284–289 (2011). 21460286 10.1093/cid/cir043 PMC3106235

[4] Girndt, M., Sester, U., Sester, M., Kaul, H. & Köhler, H. Impaired cellular immune function in patients with end-stage renal failure. *Nephrol. Dial Transpl.* **14**, 2807–2810 (1999). 10.1093/ndt/14.12.2807 10570074

[5] Hielscher, F. et al. The inactivated herpes zoster vaccine HZ/su induces a varicella zoster virus specific cellular and humoral immune response in patients on dialysis. *EBioMedicine* **108**, 105335 (2024). 39265505 10.1016/j.ebiom.2024.105335 PMC11416227

[6] Sester, U. et al. Serial influenza-vaccination reveals impaired maintenance of specific T-cell memory in patients with end-stage renal failure. *Vaccine* **31**, 4111–4120 (2013). 23845814 10.1016/j.vaccine.2013.06.076

[7] Bronder, S. et al. Potent induction of humoral and cellular immunity after bivalent BA.4/5 mRNA vaccination in dialysis patients. *NPJ Vaccines*. **9**, 25 (2024). 38326340 10.1038/s41541-024-00816-0 PMC10850212

[8] Cossmann, A. et al. Immune responses following BNT162b2 XBB.1.5 vaccination in patients on haemodialysis in Germany. *Lancet Infect. Dis.* **24**, e145–e146 (2024). 38211602 10.1016/S1473-3099(23)00783-1

[9] Davis-Gardner, M. E. et al. Neutralization against BA.2.75.2, BQ.1.1, and XBB from mRNA Bivalent Booster. *N Engl. J. Med.* **388**, 183–185 (2023). 36546661 10.1056/NEJMc2214293 PMC9812288

[10] Affeldt, P. et al. Neutralizing response against SARS-CoV-2 Omicron BA.5 and XBB.1.5 in hemodialysis patients. *Clin. Kidney J.* **16**, 2757–2759 (2023). 38046037 10.1093/ckj/sfad230 PMC10690076

[11] Koch, J. et al. Empfehlung der STIKO zur Implementierung der COVID-19-Impfung in die Empfehlungen der STIKO 2023 und die dazugehörige wissenschaftliche Begründung. *Epid Bull.* **21**, 7–48 (2023).

[12] Dulfer, E. A. et al. Timing and sequence of vaccination against COVID-19 and influenza (TACTIC): a single-blind, placebo-controlled randomized clinical trial. *Lancet Reg. Health Eur.* **29**, 100628 (2023). 37261212 10.1016/j.lanepe.2023.100628 PMC10091277

[13] Radner, H. et al. Reduced immunogenicity of BNT162b2 booster vaccination in combination with a tetravalent influenza vaccination: results of a prospective cohort study in 838 health workers. *Clin. Microbiol. Infect.* **29**, 635–641 (2023). 36509374 10.1016/j.cmi.2022.12.008

[14] Lazarus, R. et al. Safety and immunogenicity of concomitant administration of COVID-19 vaccines (ChAdOx1 or BNT162b2) with seasonal influenza vaccines in adults in the UK (ComFluCOV): a multicentre, randomised, controlled, phase 4 trial. *Lancet* **398**, 2277–2287 (2021). 34774197 10.1016/S0140-6736(21)02329-1 PMC8585490

[15] Izikson, R. et al. Safety and immunogenicity of a high-dose quadrivalent influenza vaccine administered concomitantly with a third dose of the mRNA-1273 SARS-CoV-2 vaccine in adults aged ≥ 65 years: a phase 2, randomised, open-label study. *Lancet Respir Med.* **10**, 392–402 (2022). 35114141 10.1016/S2213-2600(21)00557-9 PMC8803382

[16] Kommission für Hygiene und Infektionsprävention der DGfN. Empfehlung zu Impfmaßnahmen gegen SARS-CoV-2 und Influenza für Patienten mit chronischen Nierenkrankheiten. (2023). https://www.dgfn.eu/bekanntmachungen-details/empfehlung-zu-impfmassnahmen-gegen-sars-cov-2-und-influenza-fuer-patienten-mit-chronischen-nierenkrankheiten.html.

[17] STIKO. Empfehlungen der Ständigen Impfkommission (STIKO) beim Robert Koch-Institut 2025. *Epid Bull* **1–75** (2025).

[18] Stapic, M., Schulz, R. S., Tamayo-Cuartero, E., Kurth, T. & Brinks, R. Measuring the disease burden of seasonal influenza in Germany 2015–2020 using the incidence-based disability-adjusted life years (DALYs). *BMC Infect. Dis.* **25**, 413 (2025). 40141014 10.1186/s12879-025-10613-2 PMC11948870

[19] Speer, C. et al. Early Humoral Responses of Hemodialysis Patients after COVID-19 Vaccination with BNT162b2. *Clin. J. Am. Soc. Nephrol.* **16**, 1073–1082 (2021). 34031181 10.2215/CJN.03700321 PMC8425619

[20] Simon, B. et al. Haemodialysis patients show a highly diminished antibody response after COVID-19 mRNA vaccination compared with healthy controls. *Nephrol. Dial Transpl.* **36**, 1709–1716 (2021). 10.1093/ndt/gfab179 PMC8194560 33999200

[21] Espi, M. et al. The ROMANOV study found impaired humoral and cellular immune responses to SARS-CoV-2 mRNA vaccine in virus-unexposed patients receiving maintenance hemodialysis. *Kidney Int.* **100**, 928–936 (2021). 34284044 10.1016/j.kint.2021.07.005 PMC8286235

[22] Hossen, M. M. et al. Current understanding of CTLA-4: from mechanism to autoimmune diseases. *Front. Immunol.* **14**, 1198365 (2023). 37497212 10.3389/fimmu.2023.1198365 PMC10367421

[23] Ledo, A. et al. Elite athletes on regular training show more pronounced induction of vaccine-specific T-cells and antibodies after tetravalent influenza vaccination than controls. *Brain Behav. Immun.* **83**, 135–145 (2020). 31580932 10.1016/j.bbi.2019.09.024

[24] Schmidt, T. et al. CD4 + T-cell immunity after pandemic influenza vaccination cross-reacts with seasonal antigens and functionally differs from active influenza infection. *Eur. J. Immunol.* **42**, 1755–1766 (2012). 22585549 10.1002/eji.201242393

[25] Stankov, M. V. et al. Humoral and cellular immune responses following BNT162b2 XBB.1.5 vaccination. *Lancet Infect. Dis.* **24**, e1–e3 (2024). 37995739 10.1016/S1473-3099(23)00690-4

[26] Urschel, R. et al. SARS-CoV-2-specific cellular and humoral immunity after bivalent BA.4/5 COVID-19-vaccination in previously infected and non-infected individuals. *Nat. Commun.* **15**, 3077 (2024). 38594497 10.1038/s41467-024-47429-8 PMC11004149

[27] Marking, U. et al. Humoral immune responses to the monovalent XBB.1.5-adapted BNT162b2 mRNA booster in Sweden. *Lancet Infect. Dis.* **24**, e80–e81 (2024). 38190833 10.1016/S1473-3099(23)00779-X

[28] Chalkias, S. et al. Interim Report of the Reactogenicity and Immunogenicity of Severe Acute Respiratory Syndrome Coronavirus 2 XBB-Containing Vaccines. *J. Infect. Dis.* **230**, e279–e286 (2024). 38349280 10.1093/infdis/jiae067 PMC11326827

[29] Carreno, J. M. et al., *XBB.1.5 monovalent vaccine induces lasting cross-reactive responses to SARS-CoV-2 variants such as HV.1 and JN.1, as well as SARS-CoV-1, but elicits limited XBB.1.5 specific antibodies. mBio, e0360724* (2025). 10.1128/mbio.03607-24 PMC11980561 40042313

[30] Debisarun, P. A. et al. Induction of trained immunity by influenza vaccination - impact on COVID-19. *PLoS Pathog*. **17**, e1009928 (2021). 34695164 10.1371/journal.ppat.1009928 PMC8568262

[31] Moorlag, S., Arts, R. J. W., van Crevel, R. & Netea, M. G. Non-specific effects of BCG vaccine on viral infections. *Clin. Microbiol. Infect.* **25**, 1473–1478 (2019). 31055165 10.1016/j.cmi.2019.04.020

[32] Netea, M. G. et al. Trained Immunity: a Tool for Reducing Susceptibility to and the Severity of SARS-CoV-2 Infection. *Cell* **181**, 969–977 (2020). 32437659 10.1016/j.cell.2020.04.042 PMC7196902

[33] Shafiee, A. et al. Herpesviruses reactivation following COVID-19 vaccination: a systematic review and meta-analysis. *Eur. J. Med. Res.* **28**, 278 (2023). 37559096 10.1186/s40001-023-01238-9 PMC10413536

[34] Orru, S. et al., *Skin manifestations after immunisation with an adjuvanted recombinant zoster vaccine, Germany, 2020. Euro Surveill* ***28*** (2023). 10.2807/1560-7917.ES.2023.28.50.2300261 PMC10831415 38099347

[35] Xie, C., Yao, R. & Xia, X. The advances of adjuvants in mRNA vaccines. *NPJ Vaccines*. **8**, 162 (2023). 37884526 10.1038/s41541-023-00760-5 PMC10603121

[36] Roman, F. et al. Adjuvant system AS01: from mode of action to effective vaccines. *Expert Rev. Vaccines*. **23**, 715–729 (2024). 39042099 10.1080/14760584.2024.2382725

[37] Pulendran, B., O’Hagan, D. T. & P, S.A. & Emerging concepts in the science of vaccine adjuvants. *Nat. Rev. Drug Discov*. **20**, 454–475 (2021). 33824489 10.1038/s41573-021-00163-y PMC8023785

[38] Yau, K. et al. Differences in mRNA-1273 (Moderna) and BNT162b2 (Pfizer-BioNTech) SARS-CoV-2 vaccine immunogenicity among patients undergoing dialysis. *CMAJ* **194**, E297–E305 (2022). 35115375 10.1503/cmaj.211881 PMC9053976

[39] Karakizlis, H. et al. Immunogenicity and reactogenicity of homologous mRNA-based and vector-based SARS-CoV-2 vaccine regimens in patients receiving maintenance dialysis. *Clin. Immunol.* **236**, 108961 (2022). 35227871 10.1016/j.clim.2022.108961 PMC8875769

[40] Stumpf, J. et al. Risk of strong antibody decline in dialysis and transplant patients after SARS-CoV-2mRNA vaccination: Six months data from the observational Dia-Vacc study. *Lancet Reg. Health Eur.* **17**, 100371 (2022). 35434688 10.1016/j.lanepe.2022.100371 PMC8995854

[41] Sallusto, F., Lanzavecchia, A., Araki, K. & Ahmed, R. From vaccines to memory and back. *Immunity* **33**, 451–463 (2010). 21029957 10.1016/j.immuni.2010.10.008 PMC3760154

[42] da Silva Antunes, R. et al. Evolution of SARS-CoV-2 T cell responses as a function of multiple COVID-19 boosters. *Cell. Rep.* **44**, 115907 (2025). 40580476 10.1016/j.celrep.2025.115907 PMC12695460

[43] Molinos-Albert, L. M. et al. Long-lasting antibody B-cell responses to SARS-CoV-2 three years after the onset of the pandemic. *Cell. Rep.* **44**, 115498 (2025). 40173043 10.1016/j.celrep.2025.115498

[44] Bronder, S. et al., *Cellular and humoral immunogenicity of respiratory syncytial virus vaccination in solid organ transplant recipients. Am J. Transplant* (2025). 10.1016/j.ajt.2025.09.023 41062073

[45] Havlin, J. et al. Respiratory syncytial virus prefusion F3 vaccine in lung transplant recipients elicits CD4 + T cell response in all vaccinees. *Am. J. Transpl.* **25**, 1452–1460 (2025). 10.1016/j.ajt.2025.03.025 40169094

[46] Hall, V. G. et al., *Safety and immunogenicity of adjuvanted respiratory syncytial virus vaccine in high-risk transplant recipients: an interventional cohort study. Clin Microbiol. Infect* (2025). 10.1016/j.cmi.2025.09.013 41016596

[47] Radun, R. et al., *Immunogenicity and reactogenicity of the adjuvanted respiratory syncytial virus vaccine in patients with chronic kidney disease. Clin Kidney J in press* (2025). 10.1093/ckj/sfag093 PMC13146607 42100713

[48] Schmidt, T. et al. Immunogenicity and reactogenicity of heterologous ChAdOx1 nCoV-19/mRNA vaccination. *Nat. Med.* **27**, 1530–1535 (2021). 34312554 10.1038/s41591-021-01464-w PMC8440177

[49] Schmidt, T. et al. Cellular immunity predominates over humoral immunity after homologous and heterologous mRNA and vector-based COVID-19 vaccine regimens in solid organ transplant recipients. *Am. J. Transpl.* **21**, 3990–4002 (2021). 10.1111/ajt.16818 PMC8652989 34453872

