# Supplementary material for: SARS-CoV-2-specific immunity after XBB.1.5 vaccination is not significantly altered by subsequent influenza vaccination in dialysis patients
Source: Sci Rep. 2026 Jul 6;16:20771. doi: 10.1038/s41598-026-60726-0 (PMC13338216; doi:10.1038/s41598-026-60726-0)
Supplement: Supplementary file 1 — Supplementary Material 1 [file 41598_2026_60726_MOESM1_ESM.pdf]

Supplement

SARS-CoV-2-specific immunity after XBB.1.5 vaccination is not significantly altered by subsequent influenza vaccination in dialysis patients

Saskia Bronder, MSc[1], Rebecca Urschel, MSc[1], Felix Reinhardt[2], Janine Mihm, MD[3], Élora Schlienger, BSc[1], Tina Schmidt, PhD[1], Susanne Brückner[4], Urban Sester, MD[3], and Martina Sester, PhD[1;5;*]

[1]Department of Transplant and Infection Immunology, PharmaScienceHub, Saarland University, Homburg, Germany; [2]Dialysezentrum Saarlouis, Saarlouis, Germany; [3]Westpfalzklinikum Kaiserslautern, Kaiserslautern, Germany; [4]Arbeitsgemeinschaft Heimdialyse Saar e.V., Völklingen, Germany; [5]Center for Gender-specific Biology and Medicine (CGBM), Saarland University, Homburg, Germany.

The supplement contains 5 supplementary tables S1-S5 and 4 supplementary figures S1-S4.

Supplementary tables

Supplementary table S1. Demographic and clinical characteristics of the subgroup of dialysis patients receiving monovalent XBB.1.5 vaccination (Part I).

Characteristics		Dialysis patients (n=27)
Years of age, mean (SD)		66 (14.6)
Sex, n (%)[a]		
	Female	7 (25.9)
	Male	20 (74.1)
Type of dialysis, n (%)		
	Hemodialysis	27 (100)
	Peritoneal dialysis	0 (0)
Time on dialysis (years), mean (SD)		5.5 (5.8)
Cause of kidney failure, n (%)		
	Autoimmune-mediated nephropathy	3 (11.1)
	Chronic glomerulonephritis	4 (14.8)
	Secondary chronic renal disease	14 (51.9)
	Innate	4 (14.8)
	Other	2 (7.4)
	Unknown	0 (0)
Previous kidney transplantation, n (%)		2 (7.4)
Immunosuppressive therapy, n (%)		
	Glucocorticoids, n (%)	1 (3.7)
	MMF, n (%)	0 (0)
	Prednisolone/MMF, n (%)	0 (0)
**SARS-CoV-2-related characteristics before XBB.1.5-vaccination**		
History of COVID-19 vaccination regimen, n (%)		
	mRNA only	23 (85.2)
	Vector/mRNA combination	3 (11.1)
	unknown	1 (3.7)
Prior SARS-CoV-2-infection[b], n (%)		24 (88.9)
Total number of prior immunisation events[c], n (%)		
	3	1 (3.7)
	4	11 (40.7)
	5	12 (44.4)
	6	3 (11.1)
Type of previous immunisation, n (%)		
	vaccination	15 (55.6)
	infection	5 (18.5)
	unknown	7 (25.9)
Days since last known previous immunisation event, median (IQR)		
	vaccination	359 (57)
	infection	286 (73)

[a]Information on sex was based on individual self-declaration; [b]3 patients were assigned as previously infected based on NCAP-IgG positivity despite no known history of infection; [c]immunisation events including vaccination and infection. Abbreviations: COVID-19, coronavirus disease 2019; IQR, interquartile range; MMF, mycophenolate mofetil; SARS-CoV-2, severe acute respiratory syndrome coronavirus 2; SD, standard deviation.

Supplementary table S2. Demographic and clinical characteristics of the subgroup of dialysis patients receiving quadrivalent influenza vaccination (Part II).

Characteristics		Dialysis patients (n=26)
Years of age, mean (SD)		70.8 (14.3)
Sex, n (%)[a]		
	Female	6 (23.1)
	Male	20 (76.9)
Type of dialysis, n (%)		
	Hemodialysis	26 (100)
	Peritoneal dialysis	0 (0)
Time on dialysis (years), mean (SD)		5.5 (5.9)
Cause of kidney failure, n (%)		
	Autoimmune-mediated nephropathy	3 (11.5)
	Chronic glomerulonephritis	2 (7.7)
	Secondary chronic renal disease	15 (57.7)
	Innate	5 (19.2)
	Other	1 (3.8)
	Unknown	0 (0)
Previous kidney transplantation, n (%)		2 (7.7)
Immunosuppressive therapy, n (%)		
	Glucocorticoids, n (%)	2 (7.7)
	MMF, n (%)	
	Prednisolone/MMF, n (%)	1 (3.8)

[a]Information on sex was based on individual self-declaration; Abbreviations: MMF, mycophenolate mofetil; SD, standard deviation.

Supplementary table S3. Demographic characteristics of patients receiving either monovalent XBB.1.5 vaccination only or quadrivalent influenza vaccination only or sequential administration of both vaccines (part III).

Characteristics		XBB only (n=17)	Flu only (n=11)	XBB/Flu (n=28)	p value
Years of age, mean (SD)		65.1 (14.5)	74.8 (13.3)	70 (13.2)	0.187[d]
Sex, n (%)[a]					
	Female	6 (35.3)	4 (36.4)	5 (17.9)	0.302[e]
	Male	11 (5.9)	7 (63.6)	23 (82.1)	
Type of dialysis, n (%)					
	Hemodialysis	16 (94.1)	11 (100)	28 (100)	0.500[e]
	Peritoneal dialysis	1 (5.9)	0 (0)	0 (0)	
Time on dialysis (years), mean (SD)		3.2 (2.6)	3 (2.7)	5 (5.9)	0.321[d]
Cause of kidney failure, n (%)					
	Autoimmune-mediated nephropathy	1 (5.9)	0 (0)	4 (14.3)	
	Chronic glomerulonephritis	2 (11.8)	0 (0)	2 (7.1)	
	Secondary chronic renal disease	8 (47.1)	7 (63.6)	13 (46.4)	
	Innate	1 (5.9)	3 (27.3)	6 (21.4)	
	Other	5 (29.4)	0 (0)	1 (3.6)	
	Unknown	0 (0)	1 (9.1)	2 (7.1)	
Previous kidney transplantation, n (%)		1 (5.9)	0 (0)	3 (10.7)	
Immunosuppressive therapy, n (%)					
	Glucocorticoids, n (%)	0 (0)	1 (9.1)	3 (10.7)	
	MMF, n (%)	1 (5.9)	0 (0)	0 (0)	
	Prednisolone/MMF, n (%)	0 (0)	1 (9.1)	0 (0)	
**SARS-CoV-2-related characteristics before XBB.1.5-vaccination**					
History of COVID-19 vaccination regimen, n (%)					0.178[e]
	mRNA only	12 (70.6)	7 (63.6)	24 (85.7)	
	Vector/mRNA combination	1 (5.9)	0 (0)	2 (7.1)	
	unknown	4 (23.5)	4 (36.4)	2 (7.1)	
Prior SARS-CoV-2-infection[b], n (%)		15 (88.2)	7 (63.6)	22 (78.6)	0.318[e]
Total number of prior immunisation events[c], n (%)					0.294[e]
	3	1 (5.9)	2 (18.2)	3 (10.7)	
	4	5 (29.4)	0 (0)	9 (32.1)	
	5	6 (35.3)	3 (27.3)	10 (35.7)	
	6	2 (11.8)	2 (18.2)	4 (14.3)	
	7	0 (0)	1 (9.1)		
	unknown	3 (17.6)	3 (27.3)	2 (7.1)	
Type of previous immunisation, n (%)					0.766[e]
	vaccination	9 (52.9)	4 (36.4)	15 (53.6)	
	infection	4 (23.5)	3 (27.3)	8 (28.6)	

unknown	4 (23.5)	4 (36.4)	5 (5.9)	
Days since last known previous immunisation event, median (IQR)				
vaccination	398 (220.5)	528.5 (408.5)	383.5 (71.8)	0.134[d]
infection	495.5 (377.5)	80 (134)	350.5 (177.5)	0.050[d]

[a]Information on sex was based on individual self-declaration; [b]7 patients (XBB only: n=3, Flu only: n=2, XBB/Flu: n=2) were assigned as previously infected based on NCAP-IgG positivity despite no known history of infection; [c]immunisation events including vaccination and infection. Statistical analysis was performed using the Kruskal-Wallis test[d] or the Fisher´s test[e]. Abbreviations: COVID-19, coronavirus disease 2019; IQR, interquartile range; MMF, mycophenolate mofetil; SARS-CoV-2, severe acute respiratory syndrome coronavirus 2; SD, standard deviation.

Supplementary table S4. Demographic characteristics of the dialysis patients included for longitudinal assessment of vaccine-induced spike-specific immunity after sequential vaccination with XBB.1.5 vaccine followed by influenza vaccine.

Characteristics		Dialysis patients (n=20)
Years of age, mean (SD)		67.6 (13.6)
Sex, n (%)[a]		
	Female	5 (25)
	Male	15 (75)
Type of dialysis, n (%)		
	Hemodialysis	20 (100)
	Peritoneal dialysis	0 (0)
Time on dialysis (years), mean (SD)		5.8 (6.9)
Cause of kidney failure, n (%)		
	Autoimmune-mediated nephropathy	2 (10)
	Chronic glomerulonephritis	1 (5)
	Secondary chronic renal disease	10 (50)
	Innate	4 (20)
	Other	1 (5)
	Unknown	2 (10)
Previous kidney transplantation, n (%)		3 (15)
Immunosuppressive therapy, n (%)		
	Glucocorticoids, n (%)	2 (10)
	MMF, n (%)	0 (0)
	Prednisolone/MMF, n (%)	0 (0)
**SARS-CoV-2-related characteristics before XBB.1.5-vaccination**		
History of COVID-19 vaccination regimen, n (%)		
	mRNA only	17 (85)
	Vector/mRNA combination	1 (5)
	Unknown	2 (10)
Prior SARS-CoV-2-infection[b], n (%)		17 (85)
Total number of prior immunisation events[c], n (%)		
	3	0 (0)
	4	5 (25)
	5	9 (45)

	6	4 (20)
	7	0 (0)
	Unknown	2 (10)
Type of previous immunisation, n (%)		
	Vaccination	9 (45)
	Infection	6 (30)
	Unknown	5 (25)
Days since last known previous immunisation event, median (IQR)		
	Vaccination	382 (46)
	Infection	399 (298.7)

[a]Information on sex was based on individual self-declaration; [b]one patient were assigned as previously infected based on NCAP-IgG positivity despite no known history of infection; [c]immunisation events including vaccination and infection. Abbreviations: COVID-19, coronavirus disease 2019; IQR, interquartile range; MMF, mycophenolate mofetil; SARS-CoV-2, severe acute respiratory syndrome coronavirus 2; SD, standard deviation.

Supplementary table S5. Multivariable regression analyses between the three regimens.

Dependent variables		Spike IgG		Spike CD4		Spike CD8	
**Confounders**		**estimate (95% CI)**	**p-value**	**estimate (95% CI)**	**p-value**	**estimate (95% CI)**	**p-value**
Group	XBB/Flu [Ref]	1		1		1	
	XBB only	0.104 (-0.142 – 0.350)	0.400	-0.217 (-0.468 – 0.034)	0.089	-0.510 (-1.154 – 0.135)	0.118
	Flu only	-0.611 (-0.898 – -0.325))	**<0.0001**	0.554 (-0.846 – -0.261)	**0.0004**	-0.382 (-1.180 – 0.463)	0.341
age		0.0004 (-0.007 – 0.008)	0.919	-0.006 (-0.014 – 0.002)	0.113	-0.016 (-0.036 – 0.004	0.120
sex	male [Ref]	1		1		1	
	female	-0.252 (-0.499 – -0.006)	**0.045**	-0.01 (-0.262 – 0.241)	0.934	-0.290 (-0.948 – 0.368)	0.380
prior infection	yes [Ref]	1		1		1	
	no	-0.120 (-0.388 – 0.148)	0.373	-0.170 (-0.443 – 0.103)	0.217	0.094 (-0.630 – 0.817)	0.796

Shown are p-values of multivariable linear regression analyses with log(10)-transformed values; parameters refer to spike-specific IgG and spike-specific CD4 and CD8 T cells, with sequential XBB/Flu vaccination, male sex and a history of SARS-CoV-2-infection as a reference for categorical variables. Abbreviations: CI, 95% confidence interval; Flu, influenza; Ig, immunoglobulin.

Supplementary figures

Supplementary figure S1

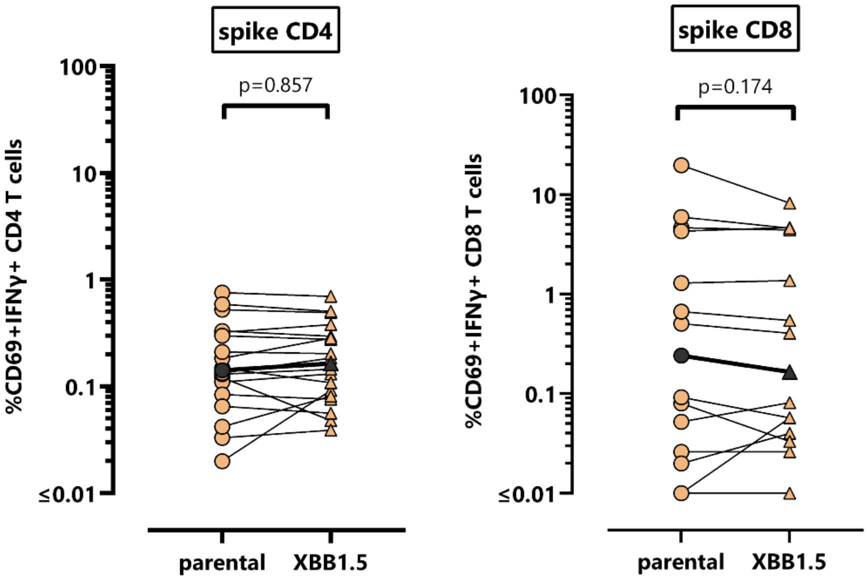

Supplementary figure S1: Comparison of CD4 and CD8 T-cell reactivity towards spike from the parental strain and the Omicron variant XBB.1.5. Levels of CD4 and CD8 T cells reactive towards parental spike (circle) and spike from the Omicron subvariant XBB.1.5 (triangle) were compared in a subgroup of patients (19 for CD4 T cells; 16 for CD8 T cells). Median levels are indicated. Differences among paired datasets were calculated by Wilcoxon signed rank test. Abbreviations: IFN, interferon.

Supplementary figure S2

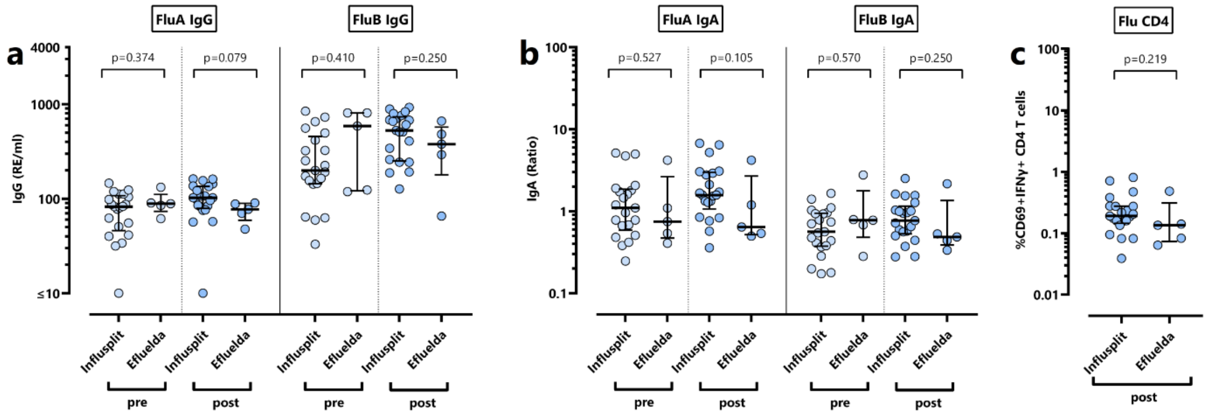

Supplementary figure S2: Comparison of influenza-specific IgG and CD4 T cells using the standard dose and high-dose influenza vaccines. Levels of **(a)** IgG towards influenza A and B, **(b)** IgA towards influenza A and B, and **(c)** influenza-specific CD4 T cells were compared in patients on the quadrivalent standard-dose vaccine (Influsplit tetra, n=21) or the high-dose vaccine (Efluelda, n=5). Median levels are indicated. Differences among paired datasets were calculated by the Mann Whitney test.

Supplementary figure S3

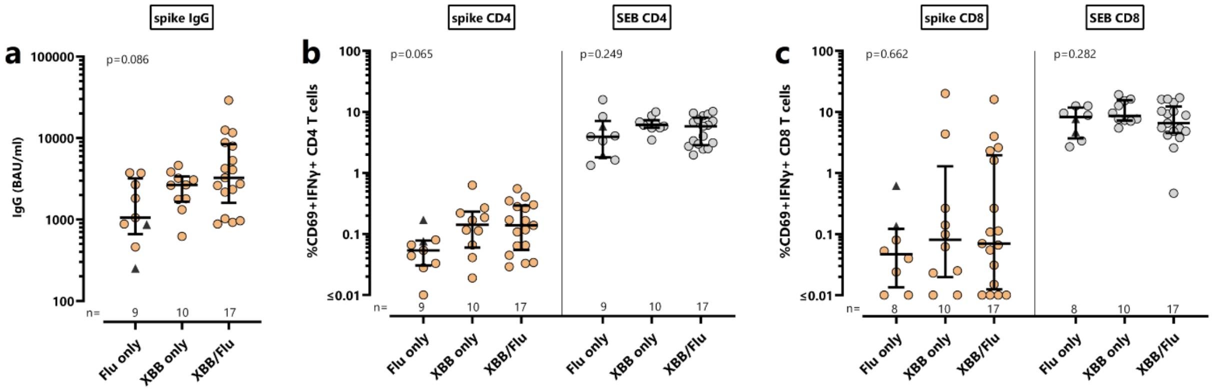

Supplementary figure S3. Comparison of baseline values of spike-specific humoral and cellular immunity in dialysis patients before sequential, monovalent XBB.1.5 alone or quadrivalent influenza vaccination alone. Levels of **(a)** spike-specific IgG antibodies, as well as **(b)** CD4 and **(c)** CD8 T cells after stimulation with peptides toward the parental spike protein or *Staphylococcus aureus* enterotoxin B (SEB) were compared between the three groups. Two patients in the Flu only group, marked by a black triangle, had a previous SARS-CoV-2 infection 20 and 56 days before administration of the influenza vaccine, respectively. Bars represent medians with interquartile ranges. Differences between groups were analysed using Kruskal-Wallis test. The number of individuals with baseline tests prior to vaccination is indicated in each panel. Abbreviations: BAU, binding antibody unit; Flu, influenza, IFN, interferon; Ig, immunoglobulin.

Supplementary figure S4

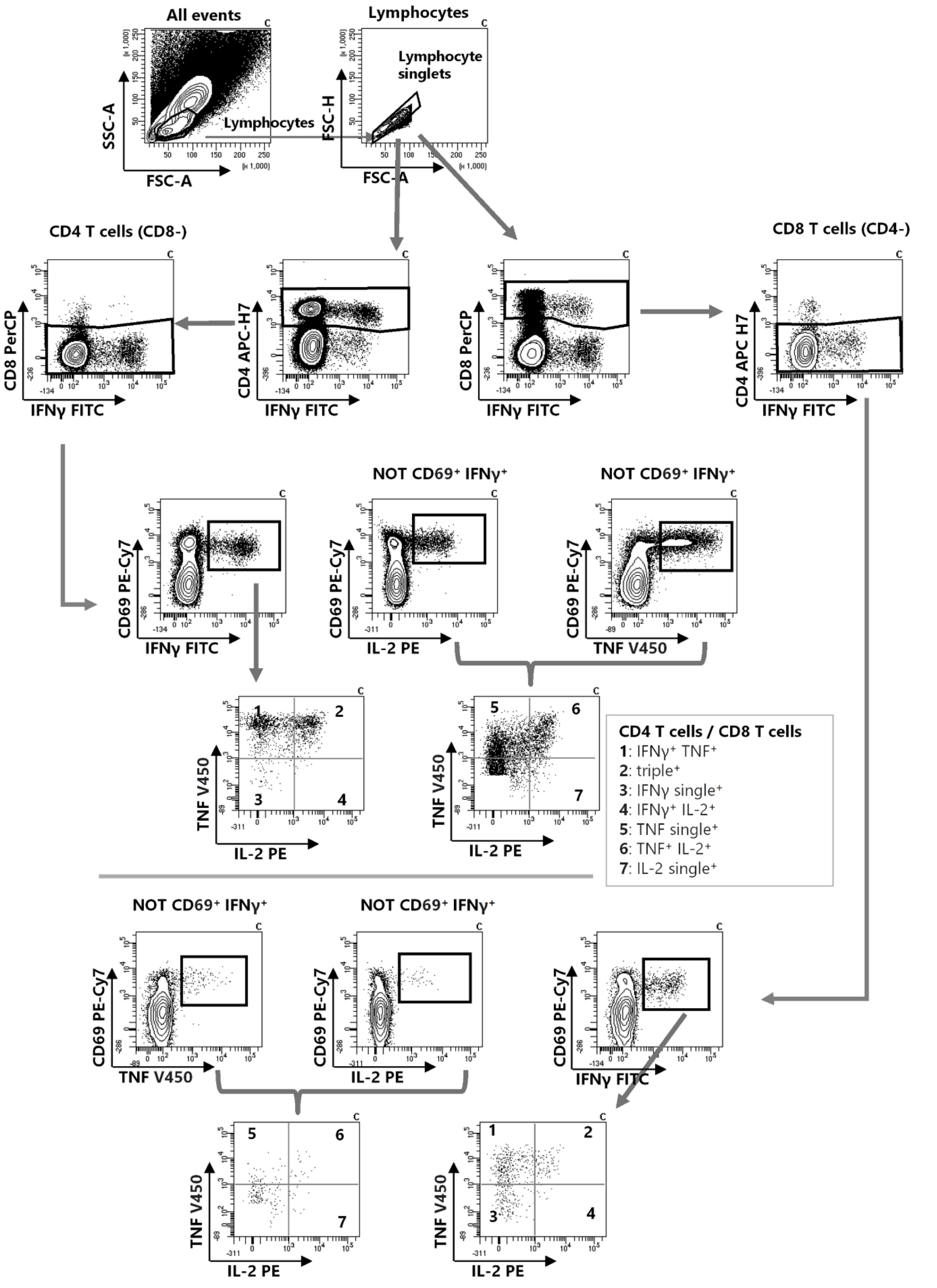

Supplementary figure S4: Gating strategy for identification of antigen-specific CD4 and CD8 T cells after stimulation. Lymphocytes were identified among total events by backgating

of CD4 and/or CD8 positive cells combined with signals for size (FSC) and granularity (SSC). Height and area signals of FSC were used to exclude doublets. The gating strategy to identify CD4 T cells or CD8 T cells co-expressing the activation marker CD69 and the cytokines IFNγ, IL-2 or TNF are shown on the upper and lower sides, respectively. Boxes were used as gates to quantify the percentage of $CD69^{+}$/$IFN\gamma^{+}$ CD4 or CD8 T cells. Moreover, Boolean gating for cytokine profiling is shown that was used to identify subpopulations of CD4 or CD8 T cells expressing all three cytokines ($triple^{+}$), two cytokines or one cytokine only.
